# Idiopathic Male Infertility Is Strongly Associated with Aberrant Promoter Methylation of Methylenetetrahydrofolate Reductase (*MTHFR*)

**DOI:** 10.1371/journal.pone.0013884

**Published:** 2010-11-09

**Authors:** Wei Wu, Ouxi Shen, Yufeng Qin, Xiaobing Niu, Chuncheng Lu, Yankai Xia, Ling Song, Shoulin Wang, Xinru Wang

**Affiliations:** 1 Key Laboratory of Reproductive Medicine, Institute of Toxicology, Nanjing Medical University, Nanjing, China; 2 Department of Urology, First Affiliated Hospital of Nanjing Medical University, Nanjing, China; CNRS, France

## Abstract

**Background:**

Abnormal germline DNA methylation in males has been proposed as a possible mechanism compromising spermatogenesis of some men currently diagnosed with idiopathic infertility. Previous studies have been focused on imprinted genes with DNA methylation in poor quality human sperms. However, recent but limited data have revealed that sperm methylation abnormalities may involve large numbers of genes or shown that genes that are not imprinted are also affected.

**Methodology/Principal Findings:**

Using the methylation-specific polymerase chain reaction and bisulfite sequencing method, we examined methylation patterns of the promoter of *methylenetetrahydrofolate reductase* (*MTHFR*) gene (NG_013351: 1538–1719) in sperm DNA obtained from 94 idiopathic infertile men and 54 normal fertile controls. Subjects with idiopathic infertility were further divided into groups of normozoospermia and oligozoospermia. Overall, 45% (41/94) of idiopathic infertile males had *MTHFR* hypermethylation (both hemimethylation and full methylation), compared with 15% of fertile controls (*P*<0.05). Subjects with higher methylation level of *MTHFR* were more likely to have idiopathic male infertility (*P*-value for trend  = 0.0007). Comparing the two groups of idiopathic infertile subjects with different sperm concentrations, a higher methylation pattern was found in the group with oligozoospermia.

**Conclusions:**

Hypermethylation of the promoter of *MTHFR* gene in sperms is associated with idiopathic male infertility. The functional relevance of hypermathylation of *MTHFR* to male fertility warrants further investigation.

## Introduction

Infertility is a worldwide reproductive health problem that affects approximately 15% of married couples. Half of these cases are due to male factors [1], and about 60%–75% of male infertility cases are idiopathic, since the molecular mechanisms underlying the defects remain unknown [2]. In these patients, oligozoospermia is frequently observed, and most infertile men resort to some kind of assisted reproductive technique (ART). Even though this method allows infertile male to father their own child without knowing the cause of their infertility, it also carries the potential risk of transmission of genetic or epigenetic aberrations to the offsprings. Because the cause of alterations in sperm production is unclear, we are interested in possible epigenetic causes.

Epigenetic alterations in DNA without concomitant changes in underlying genetic codes are now known to occur frequently in various human diseases [3]–[5]. It has been suggested that male infertility might be linked to epigenetic alterations. Previous studies have addressed this hypothesis and reported an association between abnormal DNA methylation of imprinted genes and disturbed spermatogenesis [6]–[8]. However, these studies interrogated for the most part imprinted loci with DNA methylation in poor quality human sperms. Recent but limited data [9] have revealed that sperm methylation abnormalities may involve large numbers of genes or shown that genes that are not imprinted are also affected. Although hints exist that spermatozoa from infertile men can carry aberrant DNA methylation, there is little compelling evidence to date to suggest that abnormal methylation patterns of non-imprinted genes are associated with idiopathic male infertility.

Methylenetetrahydrofolate reductase (MTHFR) is one of the main regulatory enzymes involved in folate metabolism, DNA synthesis and remethylation reactions. MTHFR catalyses the reduction of methylenetetrahydrofolate (5, 10-methyl THF) to methyltetrahydrofolate (5-methyl THF). Thus, this enzyme has a key role in balancing the pool of methyl groups between DNA synthesis and DNA methylation. Recently, several association studies have suggested that polymorphic variants in the *MTHFR* gene may be associated with reduced sperm counts in humans, leading to male infertility in some populations [10]–[13]. Additionally, a very recent study showed that *MTHFR* promoter hypermethylation was associated with non-obstructive azoospermia in testicular biopsies of patients [14]. Furthering the hypothesis that MTHFR plays an important role in spermatogenesis is the observation that MTHFR activity is about five times higher in the adult mouse testes than that in other major organs [15]. This finding, in conjunction with recent clinical evidence, suggests an important role for MTHFR in spermatogenesis.

In this study, to test our hypothesis that hypermethylation of the promoter region of *MTHFR* gene is associated with idiopathic male infertility, we utilized carefully selected fertile controls to evaluate the association between the *MTHFR* promoter hypermethylation and idiopathic male infertility.

## Materials and Methods

### Subject recruitment and sample collection

Study subjects were volunteers from the affiliated hospitals of Nanjing Medical University between March 2006 and July 2009 (NJMU Infertile Study). The study was approved by the Institutional Ethics Committee of Nanjing Medical University. All activities involving human subjects were done under full compliance with government policies and the Helsinki Declaration. Consecutive eligible men (with wives not diagnosed as infertile) were recruited to participate, 680 in total were asked. Of those approached, 82.5% consented (561 participants, 507 cases and 54 controls). Written informed consent was obtained from all study subjects. Infertile men had an infertility history of at least 2 years with their spouses with confirmed normal gynaecological assessment. The controls were healthy men who had fathered at least one healthy child within one year without assisted reproductive measures during the same period as those of the cases recruited in the same hospital. A scheduled interview was arranged for each subject to collect information, including personal background, lifestyle factors, occupational and environmental exposures, sexual and reproduction status, genetic risk factors, medical history and physical activity (e.g. exercise status). After interview, each subject donated a 5-ml peripheral blood sample and a semen sample for genetic testing. These patients and healthy donors were all ethnically Han Chinese from East China.

### Semen analysis

Semen samples were obtained in private by masturbation into a sterile widemouth and metal-free glass container after a recommended at least 3-day sexual abstinence. After liquefaction at 37°C for 30 min, conventional semen analysis was conducted in accordance with guidelines of the WHO Laboratory Manual for the Examination of Human Semen [16], including semen volume, sperm number per ejaculum, sperm concentration, motility, progression and motion parameters by using Micro-cell slide and computer-aided semen analysis (CASA, WLJY 9000, Weili New Century Science and Tech Dev.). Strict quality control measures were enforced throughout the study. Each sample was assessed twice, successively. A total of 507 infertile males underwent semen physical examination, serum determination of estradiol (E_2_), testosterone (T), prolactin (PRL), luteinizing hormone (LH), follicle-stimulating hormone (FSH), karyotyping, and molecular tests for Y-chromosome microdeletions [17], and the results of these tests excluded 413 individuals: 67 azoospermia (9 obstructive azoospermic), 23 with karyotype abnormality (nine of them with Klinefelter syndrome), 42 with Y-chromosome microdeletions, 37 with cryptorchidism and 244 secondary sterility cases. In the final analysis, we included 94 idiopathic infertile males as the patient group and 54 fertile men with normal semen parameters as the control group.

### DNA isolation

The sperms were first washed with phosphate-buffered saline twice and then washed with sperm wash buffer (SWB; 10 mM Tris-HCl, 10 mM EDTA, 1 M NaCl, pH 7.0). Motile sperm cells were purified away form lymphocyte contamination, immature germ cells and epithelial cells using swim-up method [18]. A microscopic examination of the sperm fractions was performed to control the quality of cell preparations. Sperm was placed in SWB and lysed at 55°? over night in the presence of 0.04 M dithiothreotol (DTT), 0.5 mg/ml proteinase K and 0.9% SDS [19]. DNA was extracted by using a standard method with addition of 0.1 mM 2-mercaptoethanol [20], and DNA concentration was determined by spectrophotometry.

### Methylation-Specific PCR (MSP)

Genomic DNA (1 µg, or 500 ng when sperm DNA was not enough) was treated with sodium bisulfite using the EpiTect Bisulfite Kit (QIAGEN) according to the protocol recommended by the manufacturer. Three microliters of the final eluant was used for subsequent PCR amplification. The primers used were: methylated forward: 5′-TAGATTTAGGTACGTGAAGTAGGGTAGAC-3′, methylated reverse: 5′-GAAAAACTAATAAAAAACCGACGAA-3′, unmethylated forward: 5′-TTTAGGTATGTGAAGTAGGGTAGATGT-3′, unmethylated reverse: 5′-CAAAAAACTAATAAAAAACCAACAAA-3′ [14]. For both PCR reactions, a total of 100 ng of modified DNA was amplified in a 25 µl reaction containing 0.5 µM each of forward and reverse primer, 200 µM dNTPs, 1× PCR buffer and 1.25 U of Taq Hot Start DNA Polymerase (Takara Bio, Tokyo, Japan) under the following conditions: 5 min of denaturation at 95°C followed by 40 cycles of 40 s at 95°C, 40 s at 58°C for methylated primers (59°C for unmethylated primers), 1 min at 72°C and a final extension for 10 min at 72°C. The amplification products were separated on 2% agarose gels, stained with ethidium bromide. Most samples were subject to two independent bisulfite treatments and analyzed from two independent PCR products. One control sperm DNA sample was methylated using SssI methyltransferase (New England Biolabs) according to the manufacturer's protocol and used as methylated, positive control for MSP reactions.

### Bisulfite-Sequencing PCR (BSP)

To confirm that the MSP results accurately reflected the methylation pattern of the promoter of *MTHFR*, we carried out BSP assay. We examined a total of 18 CpG sites (CpGs) in a 299 bp fragment of the promoter of *MTHFR* (NG_013351: 1525–1824; Figure 1). The primers were designed using Methprimer software (http://www.urogene.org/methprimer/index1.html). The primers used were: 5′-TGAGAAAAGATTTTAGATTTAGGTA-3′ (forward), 5′-AATCCAAAATAACAATAAAAAAAA-3′ (reverse). PCR included an initial incubation at 95°C for 10 minutes, followed by 40 cycles of 95°C for 40 seconds, 51.9°C for 40 seconds, and 72°C for 60 seconds, followed by one cycle of 72°C for 10 minutes. The PCR products were purified and cloned into the pCR2.1 vector by TA Cloning kit (Invitrogen, Carlsbad, CA, USA). To determine the methylation status of the promoter of *MTHFR* gene, an average of 15 clones (each plate) were sequenced using M13 reverse primer and an automated ABI Prism 3730xl Genetic Analyser (Applied Biosystems, Foster city, CA, USA).

**Figure 1:**
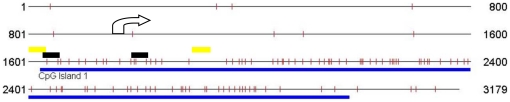
The CpG island promoter region of the *MTHFR* gene. The CpG Island Searcher Program identified a CpG island within the *MTHFR* gene promoter region, downstream of the transcriptional start site. Arrow represents the transcriptional start site, blue line depicts the CpG island and the vertical bars represent a CpG site. Black lines and yellow lines represent the location of the MSP primers and BSP primers, respectively.

### Statistical Analysis

Analysis of variance (ANOVA) was used to explore the relationships between fertility status and potentially important covariates, such as age, body mass index (BMI) and ejaculate volume. Differences in sperm concentration and motility between cases and controls were tested using Mann-Whitney test. The chi-squared test was used to evaluate the differences in smoking status, drinking status, duration of sexual abstinence, and the frequency of hypermethylation of *MTHFR* between case and control groups. To analyze the association between methylation patterns of *MTHFR* and idiopathic male infertility with normal and abnormal sperm concentrations, chi-square trend tests were performed. Logistic regression analysis was performed to obtain the odds ratios (ORs) for idiopathic male infertility and 95% confidence intervals (95% CI) with adjustment for age, BMI, smoking status and alcohol drinking, wherever it was appropriate. All statistical analyses were carried out using Stata (Version 9.0, StataCorp, LP), and *P*≤0.05 were considered to be significant.

## Results

We analyzed DNA methylation of the *MTHFR* gene in spermatozoa from 94 idiopathic infertile males and 54 age-matched fertile controls with normal endocrine status and semen parameters. Demographic categories by fertility and sperm concentration are described in Table 1.

**Table 1:** Characteristics of idiopathic infertile males and fertile controls.

Characteristic	Controls (n = 54)^a^	Cases		
		Case 1 (n = 30)^b^	Case 2 (n = 64)^c^	Case all (n = 94)^d^
Age (years, mean ± SD)	29.52±3.72	29.67±4.91	28.75±4.34	29.04±4.52
BMI (kg/m^2^, mean ± SD)^e^	22.42±2.54	21.04±4.92	23.11±2.93	22.45±3.78
Smoking status [n (%)]				
Yes	19 (35.2)	11 (36.7)	27 (42.2)	38 (40.4)
No	35 (64.8)	19 (63.3)	37 (57.8)	56 (59.6)
Alcohol drinking [n (%)]				
Yes	13 (24.1)	4 (13.3)	10 (15.6)	14 (14.9)
No	41 (75.9)	26 (86.7)	54 (84.4)	80 (85.1)
Abstinence time [n (%)]				
<4	36 (66.7)	14 (46.7)	33 (51.6)	47 (50.0)
4–7	17 (31.5)	11 (36.7)	23 (35.9)	34 (36.2)
>7	1 (1.9)	5 (16.7) *	8 (12.5)	13 (13.8)
Ejaculate volume (ml, mean ± SD)	4.24±1.04	3.11±1.16*	3.11±1.12*	3.11±1.13*
Sperm concentration (10^6^/ml)^f^	41.25 (29.22–58.14)	61.65 (40–103.5)*	9.52 (3.86–14.33)*	14.08 (5.40–36.55)*
Sperm motility (%)^f^	55.79 (44.00–67.40)	64.83 (43.48–79.21)	30.56 (16.30–41.62)*	36.03 (18.18–53.22)*

a Control: fertile men.

b Case 1: idiopathic infertile men with normozoospermia.

c Case 2: idiopathic infertile men with oligozoospermia.

d Case all: the sum of Case 1 and Case 2.

e BMI: body mass index.

f Values are given as median and interquartile range (IQR).

*P<0.05 when compared between case and control groups.

**Figure 2:**
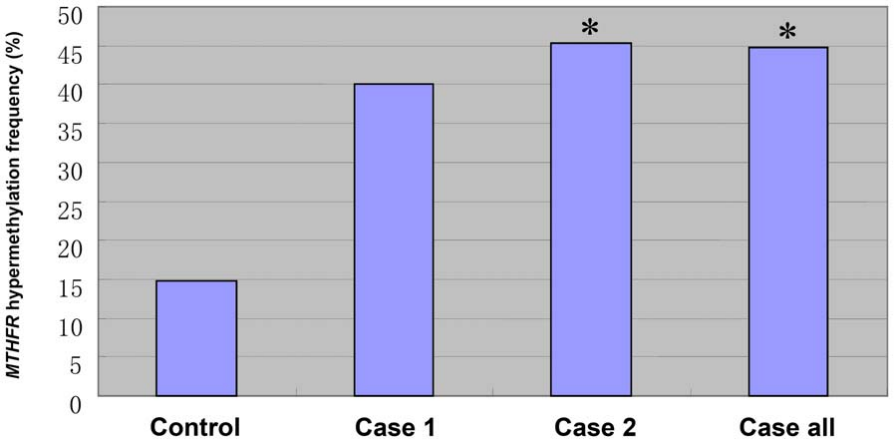
Frequencies of *MTHFR* hypermethylation (both hemimethylation and full methylation) in case and control groups. Significant differences were marked with *P<0.05 compared with the control.

**Figure 3:**
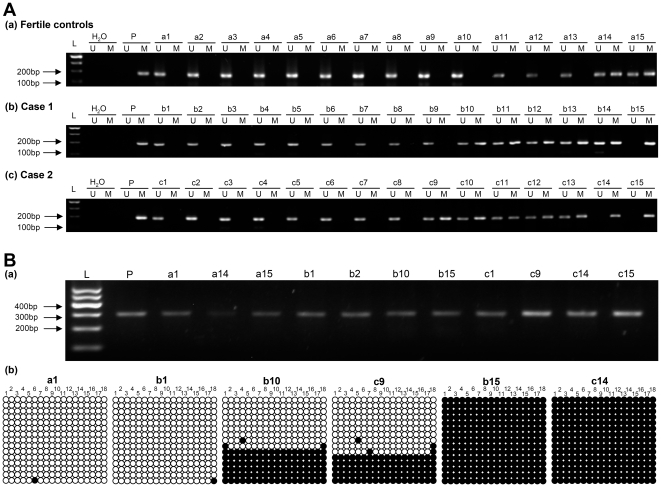
Methylation status of the promoter of *MTHFR* in genomic DNA prepared from ejaculated human sperm. (A) Representative results of the MSP analysis of *MTHFR*. DNA obtained from the sperms was amplified with primers specific to the unmethylated (U) or the methylated (M) of *MTHFR* after treatment with sodium bisulfite. (a) Fertile controls a1–a13 showed only the unmethylated allele. Others showed both methylated and unmethylated alleles. (b) Idiopathic infertile males with normozoospermia (Case 1) b1–b9 showed only the unmethylated allele. Idiopathic infertile males with normozoospermia b10–b14 showed both methylated and unmethylated alleles. Other showed only the methylated allele. (c) Idiopathic infertile males with oligozoospermia (Case 2) c1–c8 showed only the unmethylated allele. Idiopathic infertile males with oligozoospermia c9–c13 showed both methylated and unmethylated alleles. Others showed only the methylated allele. (B) Representative results of bisulfite-PCR sequencing of *MTHFR*. Filled and open circles represent methylated and unmethylated CpGs, respectively. L: molecular weight markers; P: positive control (universal methylated DNA).

Figure 1 shows a map of the CpG island (1477 bp) as well as the PCR primers utilized for methylation analysis of the promoter of *MTHFR* gene (NG_013351: 1538–1719). The percentage of hemimethylation (both methylated and unmethylated alleles) of *MTHFR* was 29% among idiopathic infertile patients and 13% among fertile controls, while the prevalence of full methylation (only the methylated allele) of *MTHFR* in idiopathic infertile males and controls were 16% and 2%, respectively. Overall, 45% (41/94) of idiopathic infertile males had hypermethylation (both hemimethylation and full methylation) in their sperms, compared with 15% of the fertile controls (Figure 2). A representative MSP analysis of these samples is shown in Figure 3A. About 5% of the samples with each methylation pattern were randomly selected and confirmed by BSP assay. The results were 100% concordant (Figure 3B). The frequency of hypermethylation of *MTHFR* was significantly higher in idiopathic infertile men than in fertile controls (*P*<0.05). These results indicate that the *MTHFR* promoter methylation was frequently altered in idiopathic infertile patients.

For a more detailed analysis, the subjects was stratified by the sperm concentration ≥20 and <20 million spermatozoa/ml into groups of 30 (Case 1) and 64 (Case 2) patients. *MTHFR* hemimethylation and full methylation was 31% and 14% respectively in Case 2 group (Table 2), the frequencies were significantly higher compared with the control group (13% and 2%, respectively; *P*<0.05). Case 1 and Case 2 groups displayed hypermethylation of 40% and 45%, respectively (Figure 2), while Case 2 were significantly different that of the controls (15%; *P*<0.05).

**Table 2:** Adjusted ORs (95% CIs) for idiopathic male infertility by methylation patterns of *MTHFR*.

Methylation	Control^a^ (n = 54)	Case					
		Case 1 (n = 30)^b^		Case 2 (n = 64)^c^		Case all (n = 94)^d^	
	n (%)	n (%)	OR (95% CI)	n (%)	OR (95% CI)	n (%)	OR (95% CI)
U^e^	46 (85.2)	18 (60.0)	1.00	35 (54.7)	1.00	53 (56.2)	1.00
U/M^f^	7 (13.0)	9 (30.0)	3.08 (0.96–9.92)	20 (31.3)	3.81 (1.41–10.31)*	29 (28.6)	3.27 (1.21–8.87)*
M^g^	1 (1.9)	3 (10.0)	6.50 (0.57–73.81)	9 (14.1)	13.21 (1.57–111.39)*	12 (16.2)	10.42 (1.30–83.19)*
*P*-value for trend			0.0191		0.0006		0.0007

a Control: fertile men.

b Case 1: idiopathic infertile men with normozoospermia.

c Case 2: idiopathic infertile men with oligozoospermia.

d Case all: the sum of Case 1 and Case 2.

e U: unmethylation (only the unmethylated allele).

f U/M: hemimethylation (both methylated and unmethylated alleles).

g M: full methylation (only the methylated allele).

*P<0.05 compared with the unmethylation of *MTHFR*.

Adjusted ORs and 95% CIs for associations between idiopathic male infertility and methylation patterns of *MTHFR* are presented in Table 2. Compared with men with only unmethylated allele, men with higher methylation levels were more likely to have idiopathic infertility [ORs for increasing methylation levels  = 1.00 for unmethylated, 3.27 (95% CI, 1.21–8.87) for hemimethylated, 9.93 (95% CI, 1.10–90.31) for full methylated; *P*-value for trend  = 0.0007]. When we divided the patients into Case 1 and Case 2 group according to a cut-off value of 20 mill./ml of the normal sperm concentration, we found an increased risk for idiopathic infertility among increasing methylation levels for both groups (*P*-value for trend  = 0.0191 and 0.0006, respectively). Comparing the two groups of idiopathic infertile subjects with different sperm concentrations, a higher methylation pattern was found in the group with oligozoospermia.

## Discussion

There is a growing body of evidence highlighting the role that aberrant DNA methylation plays in human diseases [2]–[5], [21]. Several studies of European and Japanese cohorts provided evidence that sperms from infertile men frequently had abnormal levels of DNA methylation at imprinted loci [6]–[9]. Previous studies have been focused on imprinted genes with DNA methylation in poor quality human sperms [6]–[8] and conceptuses conceived using ART (from infertile parents) [22]. However, recent but limited data have revealed that sperm methylation abnormalities may involve large numbers of genes or shown that genes that are not imprinted are also affected [9]. Houshdaran and colleagues showed that sperm abnormalities may also be associated with a broad epigenetic defect of increased DNA methylation at numerous sequences of diverse types, such as non-imprinted genes and some repetitive elements [9]. The results from these studies indicated that male infertility is associated with aberrant methylation patterns of both imprinted genes and non-imprinted genes.

Alterations in DNA methylation patterns impact several critical cellular processes including gene expression, X-inactivation, carcinogenesis, aging and development [23]–[26]. It is well established that hypomethylation is associated with transcriptional activation, whereas hypermethylation is associated with repression of transcription [23]. The product of the *MTHFR* gene is critical in maintaining an adequate methionine pool and is believed to be important in the process of DNA methylation [27]. MTHFR is crucial to the folate pathway, and disruption of this enzyme compromises mouse development in several ways [28]. In male mice, inactivation of MTHFR results in hyperhomocysteinemia and infertility with abnormal testicular histology characterized by absence of germinal cells and spermatogenesis arrest [28]. Moreover, Schwahn and coworkers postulated that accumulation of homocysteine and impaired transmethylation reactions may be the primary factors in the MTHFR-related pathology [29]. Thus, these factors may also underlie the spermatogenic failure in male infertility.

Our study has unveiled for the first time that the hypermethylation of *MTHFR* is a common event occurring in sperms of idiopathic infertile men, with 41 of 94 showing such epigenetic aberration. This rate has almost tripled the prevalence found in the fertile control group, which appears to be a frequent abnormality in idiopathic infertile men, playing a significant role in causing infertility. These results were in agreement with the findings of Houshdaran and his colleagues [9], who demonstrated striking associations between disruptive spermatogenesis and hypermethylation of several non-imptinted genes. Additionally, our results were in accordance with a more recent study [14] that suggests that the *MTHFR* promoter hypermethylation was associated with impaired spermatogenesis in infetile men. In the article by Khazamipour et al, hypermethylation of *MTHFR* was observed in testes of infertile males, suggesting that epigenetic silencing of *MTHFR* could play a role in male infertility.

Hypermethylation of the promoter of *MTHFR* gene is strongly associated with idiopathic male infertility. Therefore, analysis of promoter methylation in specific genes may provide biomarkers valuable for the identification of individuals with an elevated risk of male infertility. However, because our study is relatively small, further large studies of different populations with additional functional analysis of the hypermethylation are needed in order to elucidate the role and the mechanisms of the *MTHFR* gene in human spermatogenesis and the related pathology.
